# Supplementary material for: Cell-attribute aware community detection improves differential abundance testing from single-cell RNA-Seq data
Source: Nat Commun. 2023 Jun 5;14:3244. doi: 10.1038/s41467-023-39017-z (PMC10241145; doi:10.1038/s41467-023-39017-z)
Supplement: Supplementary file 3 — Description of Additional Supplementary Files [file 41467_2023_39017_MOESM3_ESM.pdf]

Description of Additional Supplementary Files

Supplementary Data 1: Distribution of Cd4t cell numbers among mice and age-groups.

Supplementary Data 2: Distribution of Alveolar Macrophage cell numbers among mice and age-groups.

Supplementary Data 3: Distribution of immune cells (B cell, CTL, moDC, Neu, NKT, NKT-p, nrMa, rMa, Treg) numbers among donor and disease stage (mild, severe) of COVID-19 patients.

Supplementary Data 4: Distribution of olfactory epithelium cells (sustentacular, sensory neurons, HBCs, Bowman’s gland, microvillar) numbers among donor and disease stage (normosmic, hyposmic) of post-COVID-19 patients.

Supplementary Data 5: Distribution of epithelial (Stem and Nonstem) cell numbers among donor and disease stage (N=Normal, U=unaffected FAPs, P=polyps, A=adenoma).

Supplementary Data 6: Distribution of Tcell (Tregs and NonTregs) numbers among donor and disease stage (N=Normal, U=unaffected FAPs, P=polyps, A=adenoma).
