# Supplementary material for: Cell-attribute aware community detection improves differential abundance testing from single-cell RNA-Seq data
Source: Nat Commun. 2023 Jun 5;14:3244. doi: 10.1038/s41467-023-39017-z (PMC10241145; doi:10.1038/s41467-023-39017-z)
Supplement: Supplementary file 5 — Reporting Summary [file 41467_2023_39017_MOESM5_ESM.pdf]

Corresponding author(s): Andrew E Teschendorff

Last updated by author(s): May 9, 2023

Reporting Summary

Nature Portfolio wishes to improve the reproducibility of the work that we publish. This form provides structure for consistency and transparency in reporting. For further information on Nature Portfolio policies, see our Editorial Policies and the Editorial Policy Checklist.

Statistics

For all statistical analyses, confirm that the following items are present in the figure legend, table legend, main text, or Methods section.

n/a	Confirmed	
☐	☒	The exact sample size (*n*) for each experimental group/condition, given as a discrete number and unit of measurement
☐	☒	A statement on whether measurements were taken from distinct samples or whether the same sample was measured repeatedly
☐	☒	The statistical test(s) used AND whether they are one- or two-sided<br>*Only common tests should be described solely by name; describe more complex techniques in the Methods section.*
☐	☒	A description of all covariates tested
☐	☒	A description of any assumptions or corrections, such as tests of normality and adjustment for multiple comparisons
☐	☒	A full description of the statistical parameters including central tendency (e.g. means) or other basic estimates (e.g. regression coefficient) AND variation (e.g. standard deviation) or associated estimates of uncertainty (e.g. confidence intervals)
☐	☒	For null hypothesis testing, the test statistic (e.g. *F*, *t*, *r*) with confidence intervals, effect sizes, degrees of freedom and *P* value noted<br>*Give P values as exact values whenever suitable.*
☒	☐	For Bayesian analysis, information on the choice of priors and Markov chain Monte Carlo settings
☒	☐	For hierarchical and complex designs, identification of the appropriate level for tests and full reporting of outcomes
☐	☒	Estimates of effect sizes (e.g. Cohen's *d*, Pearson's *r*), indicating how they were calculated

Our web collection on statistics for biologists contains articles on many of the points above.

Software and code

Policy information about availability of computer code

Data collection	No specific software was used to download data.
Data analysis	The main software R-packages used were: Seurat_4.1.1, SeuratObject_4.1.0, igraph_1.3.3, ELVAR_0.1.5, MASS_7.3-58.2, dplyr_1.0.9, Matrix_1.5-1 and CancerStemID_1.0 . Only ELVAR_0.1.5 is unpublished and is freely available from https://github.com/aet21/ELVAR and https://doi.org/10.6084/m9.figshare.22787498

For manuscripts utilizing custom algorithms or software that are central to the research but not yet described in published literature, software must be made available to editors and reviewers. We strongly encourage code deposition in a community repository (e.g. GitHub). See the Nature Portfolio guidelines for submitting code & software for further information.

Data

Policy information about availability of data

All manuscripts must include a data availability statement. This statement should provide the following information, where applicable:

- Accession codes, unique identifiers, or web links for publicly available datasets
- A description of any restrictions on data availability
- For clinical datasets or third party data, please ensure that the statement adheres to our policy

The snRNA-Seq dataset of colon cancer progression is publicly available from GEO (www.ncbi.nlm.nih.gov/geo/) under accession number GSE201348. The TMS scRNA-Seq data is available from https://doi.org/10.6084/m9.figshare.8273102.v2 . The scRNA-Seq dataset from Chua et al was downloaded from figshare (https://doi.org/10.6084/m9.figshare.12436517). The scRNA-Seq data from Finlay et al is available from GEO under accession numbers GSE201620, GSE184117 and

GSE139522.

Human research participants

Policy information about studies involving human research participants and Sex and Gender in Research.

Reporting on sex and gender	NA (only publicly available data was analyzed and this information was already provided in the respective publications)
Population characteristics	NA (only publicly available data was analyzed and this information was already provided in the respective publications)
Recruitment	NA (only publicly available data was analyzed and this information was already provided in the respective publications)
Ethics oversight	NA (only publicly available data was analyzed and this information was already provided in the respective publications)

Note that full information on the approval of the study protocol must also be provided in the manuscript.

Field-specific reporting

Please select the one below that is the best fit for your research. If you are not sure, read the appropriate sections before making your selection.

- [x] Life sciences
- [ ] Behavioural & social sciences
- [ ] Ecological, evolutionary & environmental sciences

For a reference copy of the document with all sections, see nature.com/documents/nr-reporting-summary-flat.pdf

Life sciences study design

All studies must disclose on these points even when the disclosure is negative.

Sample size	In this work we analyze single-cell omic data available in the public domain. Sample sizes were thus predetermined by these previous studies. As shown in our work, the studies analyzed were appropriately sized, as we had power to detect differential abundance shifts in relation to aging and disease.
Data exclusions	In individual scRNA-Seq studies, specific samples were excluded based on quality-control considerations, as described in Methods section.
Replication	Our work presents a novel statistical methodology, and as such we benchmark our method against competing algorithms on scRNA-Seq datasets where cell-type specific associations of differential abundance are known or highly biologically plausible. In this context, replication refers to the demonstration that a given method outperforms another one across independent datasets. In this regard, we have compared our method to completing algorithms on 5 independent single-cell datasets, encompassing 10 independent evaluations of specific cell-types. In addition, all dataset were analyzed using two to three different preprocessing methods to demonstrate robustness.
Randomization	Not applicable, as we analyzed publicly available data.
Blinding	Not applicable, as we analyzed publicly available data.

Reporting for specific materials, systems and methods

We require information from authors about some types of materials, experimental systems and methods used in many studies. Here, indicate whether each material, system or method listed is relevant to your study. If you are not sure if a list item applies to your research, read the appropriate section before selecting a response.

Materials & experimental systems

n/a	Involved in the study
x	Antibodies
x	Eukaryotic cell lines
x	Palaeontology and archaeology
x	Animals and other organisms
x	Clinical data
x	Dual use research of concern

Methods

n/a	Involved in the study
x	ChIP-seq
x	Flow cytometry
x	MRI-based neuroimaging
